# Establishment of age- and sex-stratified normative data for the Brief Assessment of Cognition (BAC) app in healthy Japanese individuals

**DOI:** 10.1016/j.scog.2026.100464

**Published:** 2026-09-22

**Authors:** Ryotaro Kubota, Shoki Izumi, Mari S. Oba, Shohei Sanji, Ryoko Tsuno, Mako Miwa, Kentaro Takai, Hirokazu Shima, Kazuyuki Nakagome

**Affiliations:** a Department of Forensic Psychiatry, National Center Hospital of Neurology and Psychiatry, 4-1-1 Ogawa-Higashi, Kodaira, Tokyo, 187-8551, Japan; b Department of Clinical Data Science, National Center of Neurology and Psychiatry, 4-1-1 Ogawa-Higashi, Kodaira, Tokyo, 187-8551, Japan; c Department of Psychiatry, National Center Hospital of Neurology and Psychiatry, 4-1-1 Ogawa-Higashi, Kodaira, Tokyo, 187-8551, Japan; d Medical Affairs, Sumitomo Pharma Co., Ltd., 7-1, Nihonbashi 2-chome, Chuo-ku, Tokyo, 103-6012, Japan; e National Center of Neurology and Psychiatry, 4-1-1 Ogawa-Higashi, Kodaira, Tokyo, 187-8551, Japan

**Keywords:** App, Brief Assessment of Cognition in Schizophrenia, Cognition, Japanese population, Normative data, Schizophrenia, Tablet assessment

## Abstract

The Brief Assessment of Cognition in Schizophrenia (BACS) and its Japanese version are widely used instruments for the brief and efficient assessment of cognitive domains commonly impaired in schizophrenia. A tablet-administered version, the Brief Assessment of Cognition (BAC) App, has been developed; however, normative data for the BAC App have not yet been established for the general Japanese population. This cross-sectional study aimed to establish normative data for the Japanese version of the BAC App in healthy Japanese individuals. A total of 120 participants aged 12–69 years were recruited, with 10 male and 10 female participants in each of six age groups. Cognitive performance was assessed using BAC App subtests, and mean scores and standard deviations were calculated for each age- and sex-defined subgroup. Most subtests showed stable performance distributions across age groups. No notable differences between forms were observed for the Tower of London Task. In contrast, a substantial difference between forms was observed for the Verbal Memory Task, with higher recall performance for Form 3. Structural characteristics of the word lists may contribute to form-dependent differences; therefore, form-related effects should be considered when interpreting verbal memory performance. Overall, this study provides age- and sex-stratified normative data for the Japanese version of the BAC App, supporting its standardized use as a digital cognitive assessment tool.

## Highlights

• Normative data were established for the BAC App in a healthy Japanese population.
• Age- and sex-stratified reference values were generated across subtests.
• A large between-form difference was observed in the Verbal Memory Task.
• Non-verbal tasks showed stable performance across age groups and forms.
• These findings support standardized use of the Japanese version of the BAC App.

## 1 Introduction

Cognitive impairment is one of the core symptoms of schizophrenia and has a substantial impact on social function (Green et al., 2012; Vita et al., 2022). A broad range of cognitive domains is affected in schizophrenia, including attention, executive function, verbal learning, memory (including working memory), and verbal fluency, and these domains are closely associated with work-related social functioning (Green, 2016). Cognitive impairment in schizophrenia is present across the course of the illness, with evidence of deficits in premorbid phases and substantial impairment after the onset of psychosis (Keefe, 2014). Approximately 70%–75% of patients with schizophrenia have neurocognitive impairments (Silberstein and Harvey, 2019). These findings underscore the continued importance of developing more effective treatments targeting cognitive impairment in schizophrenia, as well as simple and rapid cognitive assessment methods for clinical and research use.

The Brief Assessment of Cognition in Schizophrenia (BACS) and its Japanese version were developed to enable brief and efficient assessment of cognitive domains commonly impaired in schizophrenia (Keefe et al., 2004; Kaneda et al., 2007). Age- and sex-stratified normative data have been established for both the BACS and its Japanese version (Kaneda et al., 2013) and are used to compare clinical and general populations and to standardize individual subtest scores for calculation of composite scores.

The conventional BACS is administered in a paper-and-pencil format; however, a tablet-administered version, the Brief Assessment of Cognition (BAC) App, has been developed (Atkins et al., 2017). The BAC App enables automated scoring and is expected to reduce measurement error attributable to inter-rater variability. However, according to the National Institute of Mental Health's Measurement and Treatment Research to Improve Cognition in Schizophrenia (MATRICS) initiative and its recommendation, the availability of normative data from the general population is a desirable characteristic for cognitive assessment scales (Nuechterlein et al., 2008; Kern et al., 2008). Unlike the paper-and-pencil BACS, including its Japanese version, normative data for the BAC App have not yet been established for the general Japanese population. As a consequence, composite scores for the BAC App cannot currently be calculated for Japanese individuals, creating a clinical limitation in evaluating cognitive functioning in patients with schizophrenia in a manner comparable to assessment using the Japanese version of the BACS.

In the previous validation study, which was conducted in the U.S., significant differences were observed between the BACS and the BAC App versions, particularly in the Symbol Coding Task and Token Motor Task, despite the two sharing similar overall characteristics (Atkins et al., 2017). These findings highlight the necessity of collecting Japanese normative baseline data specifically for the BAC App.

The objective of the present study was to obtain the mean and standard deviation (SD) for each BAC App subtest within age- and sex-defined groups in healthy Japanese adolescents and adults. This will enable calculation of composite scores for Japanese individuals using the BAC App, analogous to those available for the BACS.

## 2 Methods

### 2.1 Study design

This was a cross-sectional study involving healthy Japanese adolescents and adults whose estimated IQ was determined to be ≥70 based on the Japanese Adult Reading Test-25 (JART-25) assessment (Matsuoka et al., 2006; Matsuoka and Kim, 2007). The study was conducted from March 6, 2024, to March 31, 2026. Healthy volunteers were recruited between April 2024 and September 2025. The recruitment of participants was outsourced to and conducted by Buzzreach, Inc.

The study protocol was approved by the National Center of Neurology and Psychiatry Ethics Committee (IRB11000187) and was registered with the Japan Registry of Clinical Trials under the identifier jRCT1030240378 (https://jrct.mhlw.go.jp/latest-detail/jRCT1030240378). The study adhered to the Ethical Guidelines for Medical and Biological Research Involving Human Subjects. For adults, written informed consent was obtained from participants before enrollment. For participants aged ≥16 to <18 years who had graduated from junior high school, written informed consent was obtained from both the participant and their legally authorized representative before enrollment. For participants aged <16 years who had not graduated from junior high school, written informed assent was obtained from participants, along with written consent from their legally authorized representative before enrollment.

### 2.2 Participants

After obtaining written consent or assent, as applicable, participants were screened for eligibility. The full inclusion criteria were individuals with Japanese nationality aged 12 (non-elementary school) to 69 years; individuals determined to be healthy adolescents or adults based on the Structured Clinical Interview for DSM-5-Research Version (SCID-5-RV) (First et al., 2015); individuals with no history of outpatient treatment related to psychiatric disorders; and individuals from whom written informed consent or assent, as applicable, had been obtained. The exclusion criteria were individuals whose native language was not Japanese; individuals with an estimated IQ of <70 on the JART-25 (Matsuoka et al., 2006; Matsuoka and Kim, 2007); individuals deemed ineligible based on web-based screening; individuals who had taken any medications, including over-the-counter drugs, within 1 week before the date of consent; and individuals judged by the principal investigator or co-investigators to be unsuitable for participation in the study.

### 2.3 Measures

#### 2.3.1 Background variables

Background information collected included age, JART-25 total score, and years of education, recorded as the duration of formal education completed. Sex and age were used to define subgroup strata for normative analyses.

#### 2.3.2 BAC App outcomes

Cognitive performance was evaluated using the BAC App incorporating the Japanese-translated version, developed by and used with prior permission from VeraSci (currently Clario, PA, USA). BAC App subtests included the Verbal Memory Task, Digit Sequencing Task, Verbal Fluency, Symbol Coding Task, Token Motor Task, and Tower of London Task. BAC App subtest scores were calculated according to the standard scoring procedures for each BACS subtest. Analyses were conducted for the overall analysis population and for each of the 12 subgroups defined by age (12–17 years, 18–29 years, 30–39 years, 40–49 years, 50–59 years, and 60–69 years) and sex (male and female).

#### 2.3.3 BAC App administration procedure

All subtests were administered and scored according to previously published descriptions of the BACS and BAC App (Atkins et al., 2017). The BAC App was installed on a tablet and the device was handed back and forth between the examiner and the participant during administration. Each task was administered in the following fixed order: Verbal Memory Task, Digit Sequencing Task, Token Motor Task, Verbal Fluency Task, Symbol Coding Task, and Tower of London Task. All assessments were administered by trained raters. Instructions for each subtest were delivered by the in-app narrator to ensure standardized and accurate administration; if necessary, the rater replayed the instructions via touchscreen operation to ensure that participants fully understood the task requirements before initiating each task.

#### 2.3.4 Verbal Memory Task

The Verbal Memory Task assessed the cognitive domain of verbal memory and learning. Participants were presented with a list of 15 words delivered via the in-app narration and were subsequently asked to recall and report as many words as possible. The words were presented at a fixed pace by the in-app narrator, and the order of word presentation was identical across tests; the task was administered a total of five times. The total number of words correctly recalled across the five tests was summed to generate the task score. Two list forms (Forms 1 and 3) were used from a total of eight available forms.

#### 2.3.5 Digit Sequencing Task

The Digit Sequencing Task assessed the cognitive domain of working memory. Participants were presented with auditory clusters of numbers arranged in random order (e.g., 936), with the sequence length gradually increasing across tests, and were instructed to report the presented numbers in ascending order from the smallest to the largest. The tests were delivered at a fixed pace via the in-app narration. The score was calculated as the total number of tests in which all items were reported in the correct order.

#### 2.3.6 Token Motor Task

The Token Motor Task assessed the cognitive domain of motor function. Participants were presented with a virtual container and tokens displayed on the tablet screen, and they were asked to use the index fingers of both hands simultaneously to swipe tokens from the left and right sides of the tablet into a central container as quickly as possible for 60 seconds. The score was defined as the total number of tokens correctly swiped into the container.

#### 2.3.7 Verbal Fluency

The Verbal Fluency Task is composed of the Semantic Fluency Task and the Letter Fluency Task. In the Semantic Fluency Task, participants were instructed to generate as many words as possible in the category “animals” within 60 seconds. In the Letter Fluency Task, participants were asked to complete two independent trials in which they had to generate as many words as possible beginning with a specified letter. The total number of words generated was calculated separately for each fluency task, and the total scores from both tasks were combined to produce the Verbal Fluency domain score.

#### 2.3.8 Symbol Coding Task

The Symbol Coding Task assessed the cognitive domain of speed of processing. Participants were asked to assign numbers to meaningless symbols using presented keys. Numbers were entered via a digital keypad and displayed beneath the corresponding symbols. After instruction and practice, participants were asked to complete as many items as possible within 90 seconds. The score was defined as the total number of correctly completed items.

#### 2.3.9 Tower of London Task

The Tower of London Task assessed the cognitive domain of executive function, reasoning, and problem-solving. Participants were presented with two images displayed side by side on the tablet screen, each depicting a different configuration of three colored balls arranged on three pegs. Participants were instructed to determine and report the minimum number of ball movements required to make one configuration identical to the other. Participants were required to follow task rules permitting only one ball movement at a time and requiring that a ball placed on top of another ball be moved first. The score was defined as the total number of correct responses. Two alternate forms of the Tower of London Task (Forms A and B) were available and both forms were used in the present study.

### 2.4 Sample size

The target sample size was 120 participants, with 10 male and 10 female participants in each age group: 12–17 years, 18–29 years, 30–39 years, 40–49 years, 50–59 years, and 60–69 years. The planned sample size was determined based on feasibility and a previous study of the Japanese version of the BACS (Kaneda et al., 2013). To inform selection of the sample size, we evaluated the relationship between sample size and the precision of the estimated SD. Based on the theoretical SD of the sample SD derived from the chi-squared distribution, the relative error (SD of the sample SD divided by the true SD) was 34.1% at *n* = 5, 23.2% at *n* = 10, and 16.1% at *n* = 20. Because the reduction in relative error was greater between *n* = 5 and *n* = 10 than between *n* = 10 and *n* = 20, a minimum of 10 participants per sex–age stratum was selected.

### 2.5 Statistical analysis

The analysis population was defined as participants who met the eligibility criteria and had no missing data for any BAC App subtest. Continuous variables were summarized using means and SDs.

BAC App subtest scores were calculated as follows: (raw score − age- and sex-specific mean) / age- and sex-specific SD (Keefe et al., 2008). The SDs of the sum and mean of the BAC App subtest z-scores were also calculated. For the Verbal Memory Task and the Tower of London Task, tabulations were performed separately for each of the two forms used for responses, and differences with 95% confidence intervals (CIs) were calculated between forms. Based on the observed difference between forms for the Verbal Memory Task, the following additional analyses were conducted: comparison of correct response rates between forms for words presented in the same serial position; calculation of phi coefficients between pairs of words within each form, with responses coded as correct = 1 and incorrect = 0; and hierarchical agglomerative cluster analysis using Ward's method for participants aged ≥18 years, conducted separately for each form. R version 4.1 or later was used for statistical analyses. Two-sided 95% CIs were reported. No adjustments for multiplicity were made.

## 3 Results

A total of 121 individuals provided written informed consent and underwent screening for eligibility, and 121 participants met the eligibility criteria. After excluding participants with missing data, 120 participants were included in the analysis population. Among the overall 120 participants, the mean age was 38.6 years, the mean JART-25 total score was 104.7, and the mean duration of education was 13.6 years (Table 1). Within each age subgroup, no notable sex differences in age, JART-25 score, or years of education were observed.

**Table 1:**
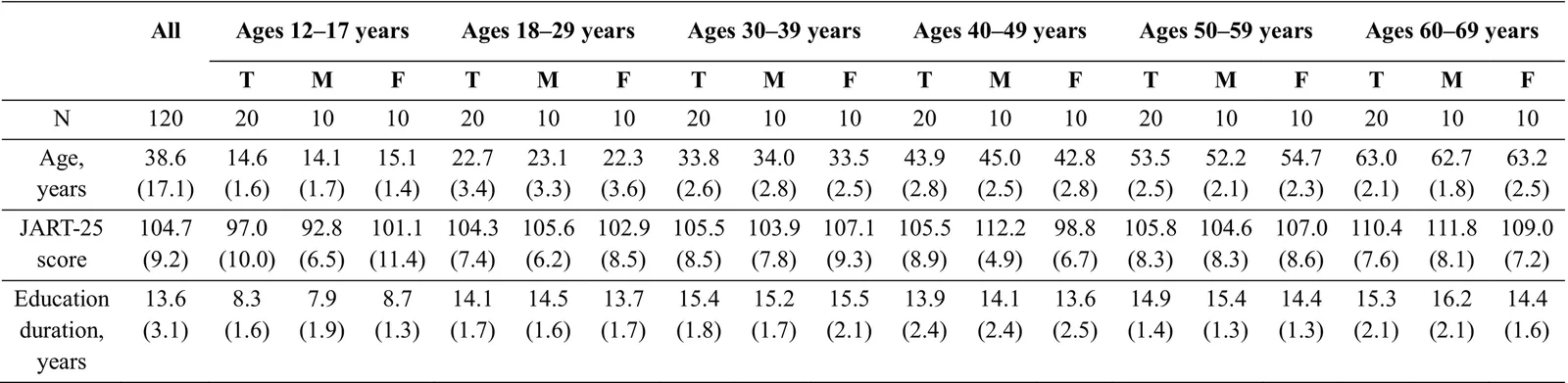
Participant characteristics.

Data are n or mean (standard deviation).

F, female; JART-25, Japanese Adult Reading Test-25; M, male; N, number of cases; T, total.

### 3.1 BAC App subtest scores

BAC App subtest scores stratified by age and sex are presented in Table 2. For the overall population, the mean (SD) scores were as follows: Verbal Memory Task, 51.36 (9.63); Digit Sequencing Task, 23.02 (3.64); Verbal Fluency Task, 46.44 (8.28); Symbol Coding Task, 55.98 (10.98); Token Motor Task, 108.47 (29.86); and Tower of London Task, 18.83 (2.06). For the Verbal Memory Task, scores were similar between participants aged 12–17 years and those in their 30s, whereas scores were numerically lower in participants in their 40s and older compared with those aged ≤30 years. For the Digit Sequencing Task, Verbal Fluency, and Tower of London Task, differences across age subgroups were relatively small, although Digit Sequencing Task and Tower of London Task scores were numerically lower in participants in their 60s. For the Symbol Coding Task and Token Motor Task, scores were numerically lower in participants in their 50s and 60s compared with younger age groups. Supplementary Table 1 shows the SDs for the sum and mean of the BAC App subtest z-scores.

**Table 2:**
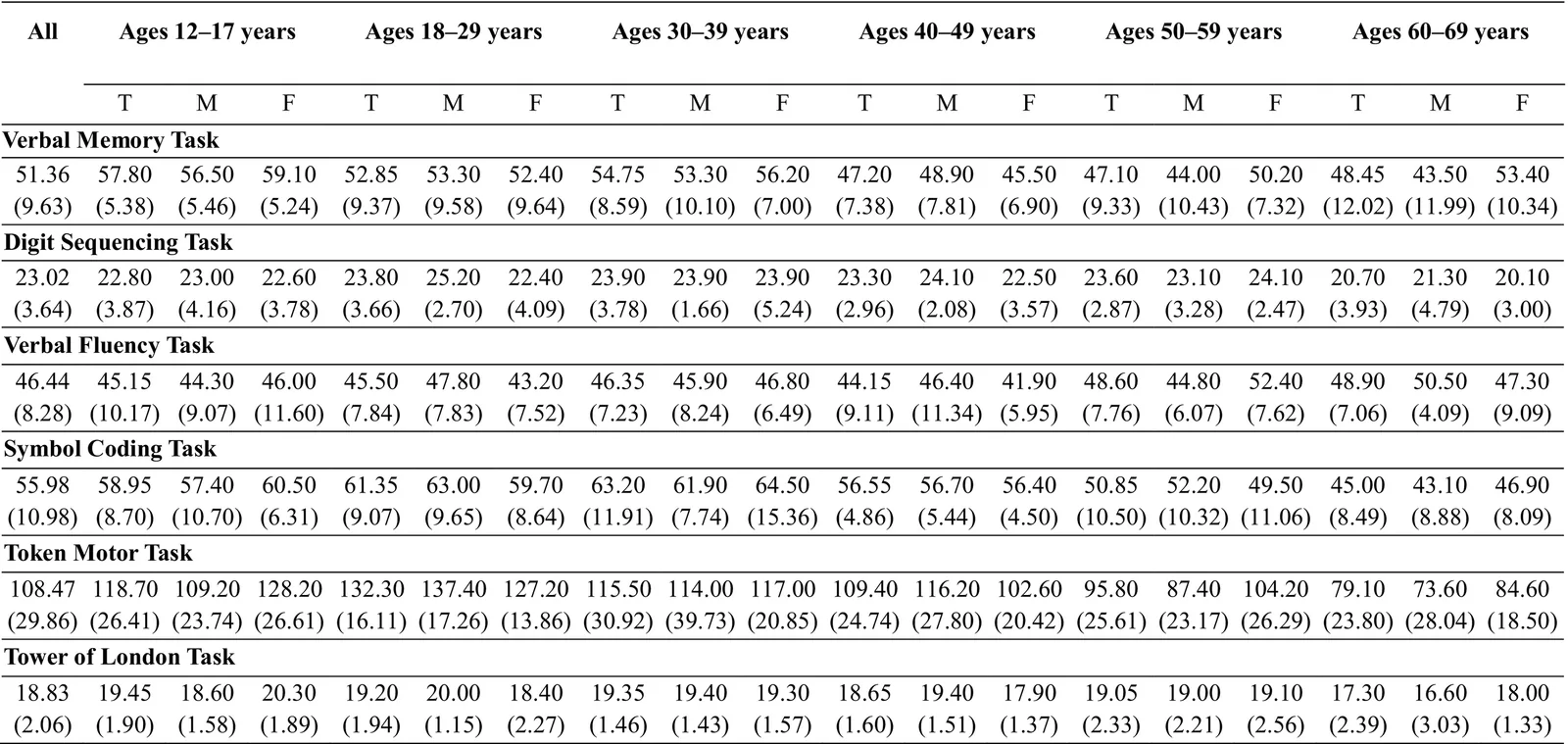
BAC App subtest scores.

Data are mean (standard deviation).

BAC App, Brief Assessment of Cognition App; F, female; M, male; T, total.

### 3.2 Differences between forms

Differences between forms for the Verbal Memory Task and Tower of London Task are presented in Table 3. For the Tower of London Task, no notable difference was observed between forms. For the Verbal Memory Task, the mean (SD) score was 46.68 (9.16) for Form 1 (*n* = 59) and 55.89 (7.77) for Form 3 (*n* = 61), with a mean difference of 9.2 (95% CI, 6.1 to 12.3). When aggregated by age group, no numerical differences between forms were observed in the 12–17 years age group, whereas a difference between forms was observed among participants aged ≥18 years (Supplementary Table 2).

**Table 3:** Differences between the two forms in the Verbal Memory Task and the Tower of London Task.

	Verbal Memory Task		Tower of London Task	
	Form 1	Form 3	Form A	Form B
Number of cases	59	61	60	60
Mean (SD)	46.68 (9.16)	55.89 (7.77)	19.08 (2.10)	18.58 (2.00)
Minimum–maximum	23–65	32–69	13–22	13–22
Mean difference (95% CI), Form 3 minus Form 1	9.2 (6.1 to 12.3)		0.5 (−0.2 to 1.2)	

CI, confidence interval; SD, standard deviation.

### 3.3 Differences in correct response rates between forms in the Verbal Memory Task

Differences in correct response rates between forms for each word in the Verbal Memory Task among individuals aged ≥18 years are shown in Fig. 1A. For all words, Form 3 showed numerically higher correct answer rates than Form 1.

**Fig. 1:**
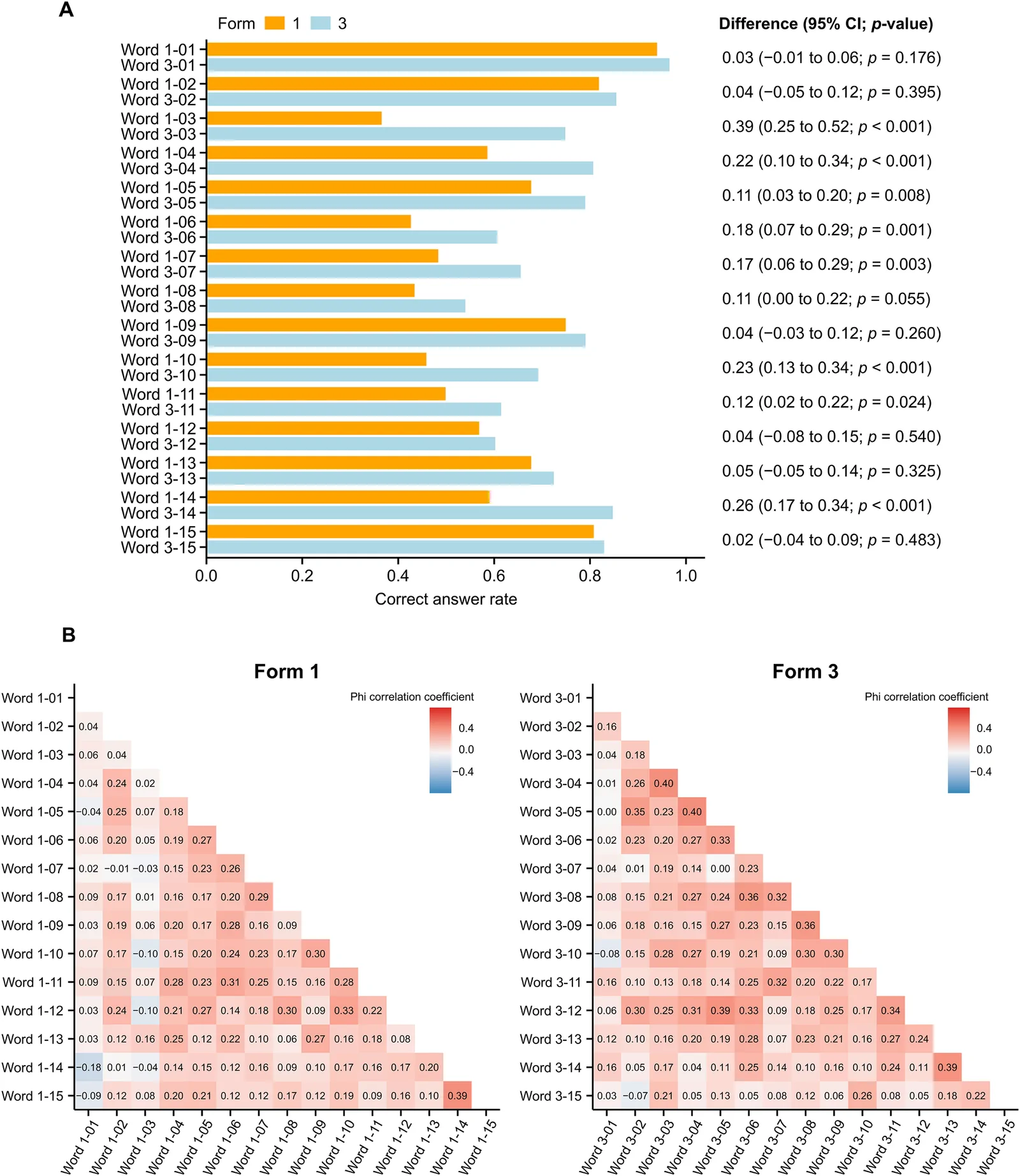

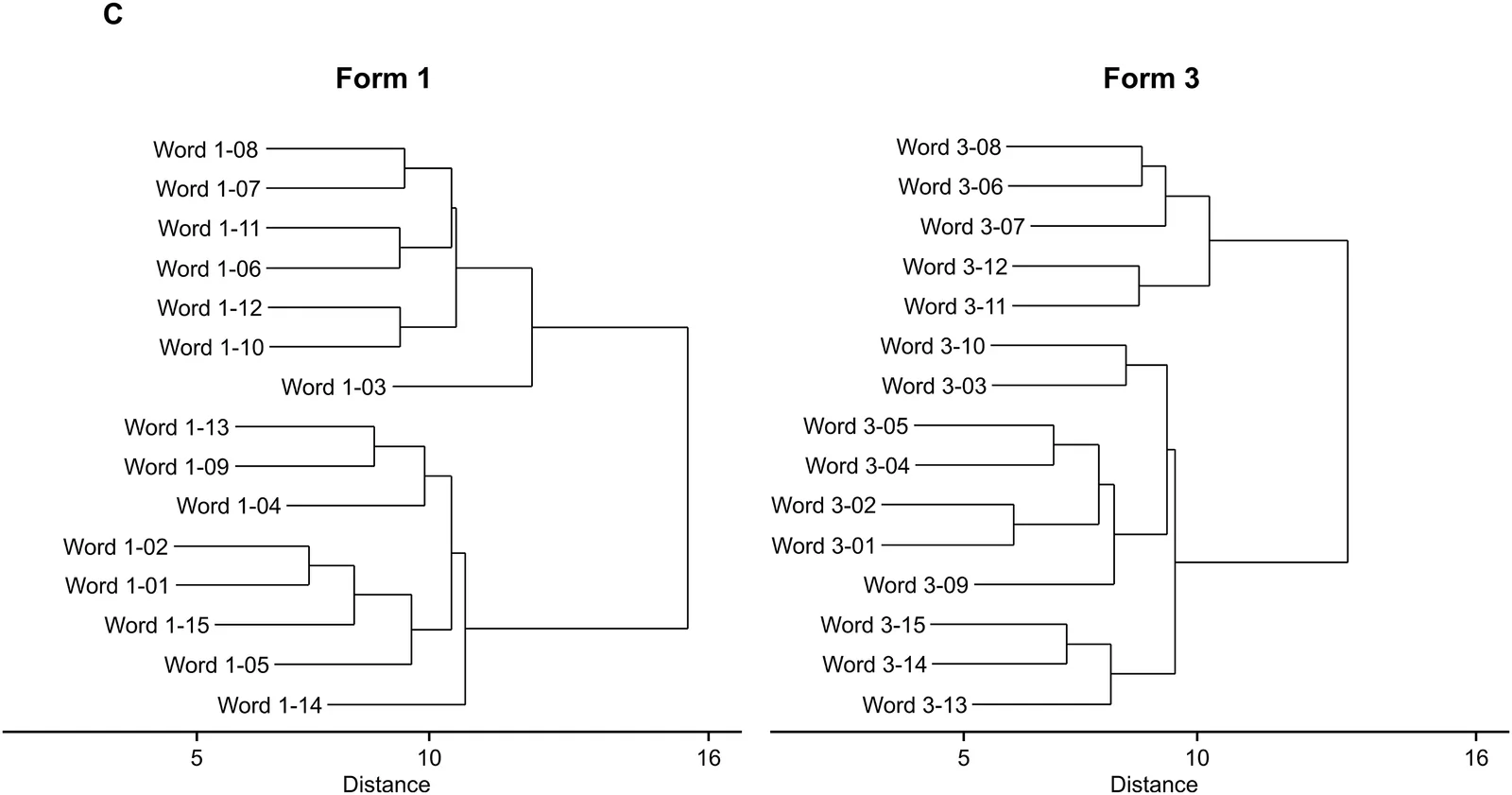
Analyses of word-level performance and relationships in the Verbal Memory Task among participants aged ≥18 years. (A) Differences in correct answer rates between forms for each word. Bars represent the proportion of correct recalls for the corresponding word in Form 1 and Form 3. Words are labeled according to their position within each list, and values indicate the proportion of correct answers. (B) Correlation coefficients of correct responses between pairs of words. Heatmaps display phi coefficients calculated for each word pair within Form 1 and Form 3, with higher values indicating stronger associations in recall patterns between words. (C) Hierarchical agglomerative cluster analysis of words. Dendrograms were generated using Ward's method based on binary recall data (correct = 1, incorrect = 0). The horizontal axis represents clustering distance, indicating the degree of dissimilarity between words, and the vertical axis shows individual words within Form 1 and Form 3. CI, confidence interval.

### 3.4 Correlation coefficients between words in the Verbal Memory Task

Correlation coefficients of correct answer rates between each word in the Verbal Memory Task for Form 1 and Form 3 among individuals aged ≥18 years are shown in Fig. 1B. In Form 1, correlation coefficients between words showed no clear adjacency pattern with respect to instruction order, and the only pair of adjacent words with a correlation coefficient exceeding 0.3 was Word 1-14 and Word 1-15 (coefficient 0.39). In Form 3, compared with Form 1, a greater number of combinations showed higher correlation coefficients between adjacent words in the instructional sequence.

### 3.5 Hierarchical agglomerative cluster analysis

The results of hierarchical agglomerative cluster analysis for each form among individuals aged ≥18 years are shown in Fig. 1C. In Form 1, two instances were observed in which the closest word pairs were adjacent in presentation order, specifically Word 1-07 and Word 1-08, and Word 1-01 and Word 1-02. In Form 3, four instances were observed in which the closest word pairs were adjacent in presentation order, specifically Word 3-11 and Word 3-12, Word 3-04 and Word 3-05, Word 3-01 and Word 3-02, and Word 3-14 and Word 3-15.

## 4 Discussion

The objective of the present study was to establish normative data for the Japanese version of the BAC App in a Japanese population. Data were collected from healthy Japanese individuals aged 12–69 years, with 20 participants (10 male and 10 female participants) included in each age group, and performance distributions and age-related characteristics were examined across BAC App subtests. For all tasks except the Verbal Memory Task, generally consistent performance patterns were observed and stable distributions were obtained. These distributions support the use of the obtained values as normative reference data for BAC App performance in Japanese individuals, enabling standardized interpretation of BAC App performance in both research and clinical contexts. These normative data provide the basis for calculating standardized BAC App scores and interpreting cognitive performance in future studies involving Japanese individuals, including investigations of cognitive impairment in schizophrenia.

Compared with previously reported values from the Japanese version of the paper-and-pencil BACS (Kaneda et al., 2013) in healthy Japanese individuals, BAC App scores in the present study were lower for the Symbol Coding Task and higher for the Token Motor Task. This finding is consistent with validation studies of the English version of the BAC App (Atkins et al., 2017), in which performance differences between the traditional BACS and the BAC App were most pronounced for the Symbol Coding Task and the Token Motor Task. The reproducibility of these differences across languages indicates that differences in administration modality between paper-and-pencil tests and tablet-based applications may influence task performance. The present study used a Japanese version of the BAC App developed through translation of the original English version. With respect to differences in performance distributions between languages, scores on the Verbal Fluency Task were higher in the validation study of the English version of the BAC App (Atkins et al., 2017). Similar cross-language differences have also been reported between the Japanese and English versions of the paper-and-pencil BACS (Keefe et al., 2004, Keefe et al., 2008; Kaneda et al., 2007). These findings highlight the importance of establishing normative data specific to the Japanese version of the BAC App and of considering appropriate adjustments in its clinical application.

In the Tower of London Task, no differences were observed between forms, whereas differences between forms were observed in the Verbal Memory Task. This result differs from findings reported for the English version of the BAC App, which showed no differences between Forms 1 and 3 (Atkins et al., 2017; Keefe et al., 2008). One possible explanation may relate to translation processes or Japanese language-specific characteristics. The Tower of London Task is largely nonverbal and thus less susceptible to language-related effects, whereas the Verbal Memory Task inherently depends on linguistic processing. International test adaptation guidelines indicate that translation alone may not ensure equivalence of psychological tests because language-specific characteristics can alter item functioning (International Test Commission, 2018). Japanese vocabulary is characterized by polysemy and numerous homophones, and when words are presented only aurally multiple semantic representations may be activated simultaneously (Chui, 2010). Word usage frequency affects lexical processing and high-frequency words are generally easier to process (Brysbaert et al., 2018). A previous study showed that frequency effects may vary depending on the task and that the influence of word frequency is not always strong in vocabulary learning (Chang and Hu, 2018). Taken together, these findings suggest that linguistic characteristics may contribute to differences in recall performance between word lists, although lexical properties such as word frequency or semantic similarity were not directly evaluated in the present study.

Analyses of inter-word correlations revealed different patterns between the two forms. In Form 1, correlations between words appeared randomly distributed and were not related to presentation order; by contrast, in Form 3, relatively higher correlations were observed between words that were adjacent in the instructional sequence. Consistent with these findings, hierarchical cluster analysis showed that pairs of words with the shortest distances were more frequently adjacent in Form 3 than in Form 1. These findings suggest that these patterns may reflect structural characteristics of the word list that could facilitate associative processing between certain items. These findings are exploratory and based solely on analyses of the present dataset. Further investigation using external linguistic resources such as large-scale language corpora is required to determine whether semantic, phonological, or usage-based relationships exist among these words.

When applying the BAC App in schizophrenia research, potential task-structure effects related to associative processing may need to be considered. Strategic memory processes are known to be impaired in schizophrenia (Guo et al., 2019). Because performance differences between forms may partly reflect the use of such processes, the differences observed in healthy individuals may manifest differently in individuals with schizophrenia. Therefore, caution is warranted when generalizing the present findings to schizophrenia populations. Future studies are needed to examine how individuals with schizophrenia engage in different forms and how this influences task performance. It is particularly important to investigate differences between Form 1 and Form 3 in schizophrenia from the perspective of strategic and associative memory processes. Findings from such studies may help to inform task design that ensures uniform availability of associative encoding across forms.

We found a tendency for age to influence cognitive function as measured by the BAC App. This association may reflect not only age-related cognitive differences but also differences in digital literacy or usability (Andias et al., 2024). This was particularly evident in the Token Motor Task. In the paper-and-pencil version of the BACS, Token Motor Task scores did not differ substantially across age groups. However, in the BAC App version, younger participants showed significantly better performance than older participants. Furthermore, scores obtained using the BAC App were generally higher than those obtained using the paper-and-pencil version. A previous study found that older adults were generally slower than younger adults during touchscreen tasks (Findlater et al., 2013). Therefore, performance on tablet-based cognitive assessments may reflect not only cognitive function itself but also differences in touchscreen operation skills. Although the present study did not assess usability, younger individuals generally demonstrated greater ease of use than older individuals. As younger generations age, the distribution of digital usability skills is likely to change over time. Therefore, it is important to periodically re-establish normative data.

The distributional characteristics obtained for tasks other than the Verbal Memory Task did not show variability that would limit their use as normative data, although further data accumulation would improve the precision of these estimates. In contrast, for the Verbal Memory Task, potential effects related to form structure should be considered when interpreting scores. In particular, caution is required when using results obtained from Forms 1 and 3 of the Verbal Memory Task in the Japanese version of the BAC App. In research applications, these findings could be used as normative data with appropriate consideration of form-related effects, such as incorporating form as a covariate or applying form-specific adjustments.

### 4.1 Limitations

The sample size was relatively small, which may have led to limited statistical power for examining structural characteristics of the Verbal Memory Task. This may also have limited evaluation of demographic factors that could affect BAC App performance, including age, years of education, and lexical experience. However, our study secured the minimum required sample size, thereby limiting the relative error. Nevertheless, estimates derived from a small sample may have larger standard errors and may inflate or deflate standardized scores when applied to patients with schizophrenia. Test–retest reliability was not evaluated, and further studies are required to examine reproducibility of BAC App scores. The study was conducted at a single center and caution is required when generalizing the results to the broader Japanese population. Future multicenter studies or pooled analyses of data from multiple institutions may enable the establishment of more representative normative datasets.

## 5 Conclusions

The present study characterized performance on the Japanese version of the BAC App across cognitive domains in a sample of healthy native Japanese speakers. Our results also highlight characteristics of task performance across forms and verbal measures that may reflect linguistic and task-structure influences in the Japanese version of the BAC App. The present study provides foundational, standardized reference data for the Japanese version of the BAC App and supports its use as a digital cognitive assessment tool in native Japanese speakers.

## CRediT authorship contribution statement

**Ryotaro Kubota:** Writing – review & editing, Writing – original draft, Investigation, Conceptualization. **Shoki Izumi:** Writing – review & editing, Writing – original draft, Visualization, Validation, Methodology, Formal analysis, Data curation, Conceptualization. **Mari S. Oba:** Writing – review & editing, Writing – original draft, Visualization, Validation, Methodology, Formal analysis, Data curation, Conceptualization. **Shohei Sanji:** Writing – review & editing, Writing – original draft, Conceptualization. **Ryoko Tsuno:** Writing – review & editing, Writing – original draft, Validation, Investigation, Data curation, Conceptualization. **Mako Miwa:** Writing – review & editing, Writing – original draft, Investigation, Data curation, Conceptualization. **Kentaro Takai:** Writing – review & editing, Writing – original draft, Funding acquisition, Conceptualization. **Hirokazu Shima:** Writing – review & editing, Writing – original draft, Funding acquisition, Conceptualization. **Kazuyuki Nakagome:** Writing – review & editing, Writing – original draft, Validation, Supervision, Project administration, Methodology, Investigation, Funding acquisition, Data curation, Conceptualization.

## Ethical approval statement

The study protocol was approved by the National Center of Neurology and Psychiatry Ethics Committee (IRB11000187) and was registered with the Japan Registry of Clinical Trials under the identifier jRCT1030240378 (https://jrct.mhlw.go.jp/latest-detail/jRCT1030240378). The study was conducted in accordance with the Ethical Guidelines for Medical and Biological Research Involving Human Subjects.

## Declaration of Generative AI and AI-assisted technologies in the writing process

During the preparation of this work, the authors used ChatGPT to improve readability and language. Professional medical writing support, independent of the use of AI, was also provided by medical writers and is acknowledged separately. After using ChatGPT and receiving professional medical writing support, the authors reviewed and edited the content as needed and take full responsibility for the content of the published article.

## Funding

This study was funded by Sumitomo Pharma Co., Ltd.

## Declaration of competing interest

Ryotaro Kubota, Shoki Izumi, Mari S. Oba, Shohei Sanji, Ryoko Tsuno, Mako Miwa, and Kazuyuki Nakagome received support for research funding, medical writing, and article processing charges from Sumitomo Pharma Co., Ltd., related to the submitted work. Ryotaro Kubota received research funding from DENTSU INC. and lecture fees from Sumitomo Pharma Co., Ltd. and Otsuka Pharmaceutical Co., Ltd., outside the submitted work. Mari S. Oba received research funding from SUSMED, Inc. and honoraria from Pfizer Japan Inc., Chugai Pharmaceutical Co., Ltd., Daiichi Sankyo Co., Ltd., and the Japan Association of Site Management Organizations, outside the submitted work. Kentaro Takai and Hirokazu Shima are employees of Sumitomo Pharma Co., Ltd. Kazuyuki Nakagome received honoraria from Sumitomo Pharma Co., Ltd., Otsuka Pharmaceutical Co., Ltd., Viatris Pharmaceuticals Japan G.K., Nippon Boehringer Ingelheim Co., Ltd., Boehringer Ingelheim International GmbH, and Yoshitomiyakuhin Corporation, and support for transportation to attend meetings from Sumitomo Pharma Co., Ltd., Otsuka Pharmaceutical Co., Ltd., and Nippon Boehringer Ingelheim Co., Ltd., outside the submitted work. His institution received research funding from SHIONOGI & Co., Ltd., Sumitomo Pharma Co., Ltd., Otsuka Pharmaceutical Co., Ltd., Janssen Pharmaceutical K.K., Nippon Boehringer Ingelheim Co., Ltd., AbbVie GK, Bristol-Myers Squibb K.K., and XNef, Inc., outside the submitted work.

## Data Availability

The data underlying this study are not publicly available. Requests for access to the data may be considered upon reasonable request to the corresponding author, subject to review and approval by the lead author and the sponsoring company.
