# Supplementary material for: Establishment of age- and sex-stratified normative data for the Brief Assessment of Cognition (BAC) app in healthy Japanese individuals
Source: Schizophr Res Cogn. 2026 Sep 22;47:100464. doi: 10.1016/j.scog.2026.100464 (PMC13677276; doi:10.1016/j.scog.2026.100464)
Supplement: Supplementary file 1 [file mmc1.docx]

**Supplementary Tables**

**Supplementary Table 1.** Sum and mean across BAC App subtest z-scores

| **All** | **Ages 12–17 years** | | | **Ages 18–29 years** | | | **Ages 30–39 years** | | | **Ages 40–49 years** | | | **Ages 50–59 years** | | | **Ages 60–69 years** | | | |
| --- | --- | --- | --- | --- | --- | --- | --- | --- | --- | --- | --- | --- | --- | --- | --- | --- | --- | --- | --- |
|  | T | M | F | T | M | F | T | M | F | T | M | F | T | M | F | T | M | F |  |
| **Sum across subtest z-scores** | | | | | | | | | | | | | | | | | | |  |
| 0.00 (3.08) | 0.00 (3.38) | 0.00 (3.72) | 0.00 (3.20) | 0.00 (3.08) | 0.00 (3.48) | 0.00 (2.81) | 0.00 (3.54) | 0.00 (3.14) | 0.00 (4.08) | 0.00 (2.28) | 0.00 (2.03) | 0.00 (2.62) | 0.00 (3.23) | 0.00 (2.91) | 0.00 (3.69) | 0.00 (3.20) | 0.00 (3.64) | 0.00 (2.89) |  |
| **Mean across subtest z-scores** | | | | | | | | | | | | | | | | | | |  |
| 0.00 (0.51) | 0.00 (0.56) | 0.00 (0.62) | 0.00 (0.53) | 0.00 (0.51) | 0.00 (0.58) | 0.00 (0.47) | 0.00 (0.59) | 0.00 (0.52) | 0.00 (0.68) | 0.00 (0.38) | 0.00 (0.34) | 0.00 (0.44) | 0.00 (0.54) | 0.00 (0.48) | 0.00 (0.62) | 0.00 (0.53) | 0.00 (0.61) | 0.00 (0.48) |  |

Data are mean (standard deviation).

BAC App, Brief Assessment of Cognition Application; F, female; M, male; T, total.

**Supplementary Table 2.** Differences between the two forms in the Verbal Memory Task according to age groups

|  | **Ages 12–17 years** | | **Ages 18–29 years** | | **Ages 30–39 years** | | **Ages 40–49 years** | | **Ages 50–59 years** | | **Ages 60–69 years** | |
| --- | --- | --- | --- | --- | --- | --- | --- | --- | --- | --- | --- | --- |
| **Verbal Memory Task** | **Form 1** | **Form 3** | **Form 1** | **Form 3** | **Form 1** | **Form 3** | **Form 1** | **Form 3** | **Form 1** | **Form 3** | **Form 1** | **Form 3** |
| Number of cases | 8 | 12 | 11 | 9 | 9 | 11 | 11 | 9 | 15 | 5 | 5 | 15 |
| Mean (SD) | 56.38  (5.66) | 58.75  (5.21) | 48.09  (9.59) | 58.67  (4.92) | 50.44  (8.85) | 58.27  (6.87) | 42.36  (5.03) | 53.11  (5.13) | 43.93  (8.40) | 56.60  (4.04) | 39.00  (9.30) | 51.60  (11.36) |
| Median (Q1, Q3) | 54.00  (53.25, 59.00) | 59.00 (53.75, 62.50) | 47.00  (41.50, 56.00) | 60.00  (56.00, 63.00) | 50.00  (43.00, 57.00) | 59.00  (52.50, 62.00) | 42.00  (40.00, 45.00) | 53.00  (52.00, 56.00) | 45.00  (37.50, 49.00) | 57.00  (53.00, 60.00) | 41.00  (40.00, 45.00) | 54.00  (43.50, 60.50) |
| Minimum–Maximum | 51–65 | 52–66 | 33–62 | 50–64 | 38–62 | 49 –69 | 34–51 | 42–60 | 29–57 | 52–61 | 23–46 | 32–68 |

Q, quartile; SD, standard deviation.
